# Effective inhibition of lytic development of bacteriophages λ, P1 and T4 by starvation of their host, *Escherichia coli*

**DOI:** 10.1186/1472-6750-7-13

**Published:** 2007-02-26

**Authors:** Marcin Łoś, Piotr Golec, Joanna M Łoś, Anna Węglewska-Jurkiewicz, Agata Czyż, Alicja Węgrzyn, Grzegorz Węgrzyn, Peter Neubauer

**Affiliations:** 1 Department of Molecular Biology, University of Gdańsk, Kładki 24, 80-822 Gdańsk, Poland; 2 Laboratory of Molecular Biology (affiliated with the University of Gdańsk), Institute of Biochemistry and Biophysics, Polish Academy of Sciences, Kładki 24, 80-822 Gdańsk, Poland; 3 Bioprocess Engineering Laboratory, Biocenter Oulu and Department of Process and Environmental Engineering, University of Oulu, P.O.Box 4300, FI-90014 Oulu, Finland; 4 Institute of Oceanology, Polish Academy of Sciences, Św. Wojciecha 5, 81-347 Gdynia, Poland

## Abstract

**Background:**

Bacteriophage infections of bacterial cultures cause serious problems in genetic engineering and biotechnology. They are dangerous not only because of direct effects on the currently infected cultures, i.e. their devastation, but also due to a high probability of spreading the phage progeny throughout a whole laboratory or plant, which causes a real danger for further cultivations. Therefore, a simple method for quick inhibition of phage development after detection of bacterial culture infection should be very useful.

**Results:**

Here, we demonstrate that depletion of a carbon source from the culture medium, which provokes starvation of bacterial cells, results in rapid inhibition of lytic development of three *Escherichia coli *phages, λ, P1 and T4. Since the effect was similar for three different phages, it seems that it may be a general phenomenon. Moreover, similar effects were observed in flask cultures and in chemostats.

**Conclusion:**

Bacteriophage lytic development can be inhibited efficiently by carbon source limitation in bacterial cultures. Thus, if bacteriophage contamination is detected, starvation procedures may be recommended to alleviate deleterious effects of phage infection on the culture. We believe that this strategy, in combination with the use of automated and sensitive bacteriophage biosensors, may be employed in the fermentation laboratory practice to control phage outbreaks in bioprocesses more effectively.

## Background

Bacteriophage infections cause serious problems in both research laboratories and large biotechnological companies [1]. Once infected by bacteriophages, bacterial cultures are usually completely destroyed, as phage lytic development in a bacterium ends up with cell lysis and liberation of progeny phages that infect neighbor bacterial cells. A possibility of spreading of bacteriophages throughout a laboratory is even more dangerous than a loss of a single culture. Subsequent cultures may be infected, which can lead to cultivation problems lasting even several months or longer. Therefore, a method for inhibition of bacteriophage lytic development in infected cultures would be very useful. Perhaps it is not difficult in small cultures (e.g. flask cultures), when simple sterilization of the whole material and a flask should be sufficient. However, phage contamination of bioreactors is a serious technical problem. Unfortunately, it is known that no matter how good the laboratory/factory practice and hygiene are, bacteriophage infections occur from time to time [2]. Although advices of how to reduce a risk for deleterious effects of bacteriophage contamination in laboratory cultures and biotechnological factories have recently been summarized [2], it appears that currently there is no method which could ensure a protection of bacterial cultures against bacteriophages.

*Escherichia coli *is one of the most widely used bacterium in genetic engineering and biotechnology. This bacterium is, however, a host for many bacteriophages and thus, it is endangered by phage infections. Bacteriophages have been considered as models in genetic and biochemical studies for a long time. However, many physiological aspects of bacteriophages' development were not sufficiently investigated relative to extensive molecular biology studies [3]. On the other hand, recent reports indicated that development of bacteriophages largely depends on the physiology of the host cells [3-5]. In laboratories, the physiological status of a cell depends on cultivation conditions. Therefore, we aimed to find cultivation conditions that may result in inhibition of lytic development of bacteriophages and that are not deleterious for bacterial cells. Previous studies indicated that development of phages T4 and λ is significantly less effective in slowly growing host cells than in rapidly growing bacteria [3-7]. Thus, we aimed to test whether induction of starvation, caused be depletion of a carbon source from the culture medium, may inhibit phage development effectively.

## Results

### Inhibition of phage development by removing of a carbon source from the culture medium in flask experiments

It was demonstrated previously that lytic development of phages λ and T4 is less efficient in slowly growing cells than in rapidly growing bacteria [3-5]. Various growth rates of bacterial cultures may be achieved by varying the carbon source in a synthetic medium. Removing of the carbon source induces starvation conditions and inhibits bacterial growth. Therefore, we asked whether removing of the carbon source can lead to inhibition of formation of progeny phages in infected bacterial cultures.

Bacteria growing in laboratory flasks in FB medium supplemented with 0.2% glucose were infected with various phages (λ, P1 or T4). After one hour (this time was sufficient for effective phage adsorption and injection of phage DNA into host cells, but this period was too short for completion of the lytic cycle in bacteria growing in a minimal medium), the infected culture was divided into two parts. Then, both cultures were centrifuged, the pellets were washed and resuspended in the same medium either containing 0.2% glucose or devoid of the carbon source. These cultures (either with or without glucose) were incubated at 37°C, and samples for phage titration were withdrawn at time intervals. At various times, the culture initially devoid of glucose was supplemented with this sugar (final concentration of 0.2%).

**Figure 1:**
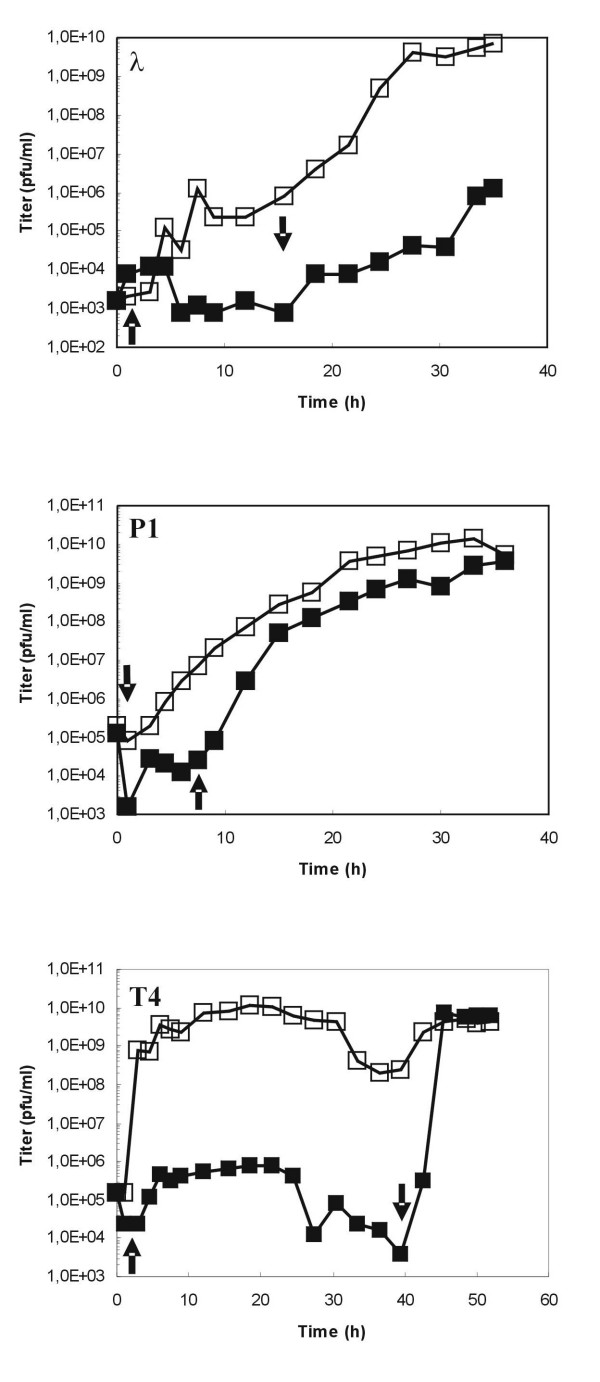
Development of bacteriophages λ (strain λ papa), P1 (strain P1*vir*) and T4 (strain T4D) in cultures of *E. coli *MG1655 growing in FB medium supplemented with 0.2% glucose (open squares) or devoid of the carbon source (closed squares). In the latter case, glucose was removed from the medium at the time indicated by the first arrow. The second arrow indicates the time of addition of glucose to the culture to final concentration of 0.2%. Bacteria were infected with phages at m.o.i (multiplicity of infection) of 0.001. Samples of cultures were withdrawn at indicated times and number of phages (plaque forming units, pfu) per ml of the culture was estimated by titration.

We found that formation of phage progeny was completely inhibited in infected cultures devoid of the carbon source (Fig. 1). This was true for all tested bacteriophages (λ, P1 and T4). Addition of glucose to infected cultures of starved bacteria resulted in restoration of phage progeny production, indicating that depletion of the carbon source was the sole reason for inhibition of development of phages λ, P1 and T4 (Fig. 1). The length of the period of starvation, after which glucose was added back to the culture, had no significant influence on restoration of bacteriophages' development, at least in the investigated range (7 – 40 hours of starvation; Fig. 1 and data not shown).

When starvation of bacteria was initiated before bacteriophage infection, no bacteriophage replication could be detected. Namely, titer of each investigated phage (λ, P1 or T4) was constant (equal to the initial titer at the infection time) and did not change significantly during the whole experiment, i.e. for several hours (data not shown). Therefore, we conclude that the investigated phages cannot develop lytically in bacterial cells starved for carbon source.

### Effects of carbon source depletion on lytic development of bacteriophages in bacteria cultured in a chemostat

Since conditions of cultivation of bacteria in laboratory flasks may differ considerably from those used in large bioreactors [8,9], we investigated effects of carbon source depletion on bacteriophage development in bacteria cultured in a chemostat.

We found that infection of *E. coli *by bacteriophage λ is inefficient under chemostat conditions, i.e. no increase in phage titer was observed (data not shown).

**Figure 2:**
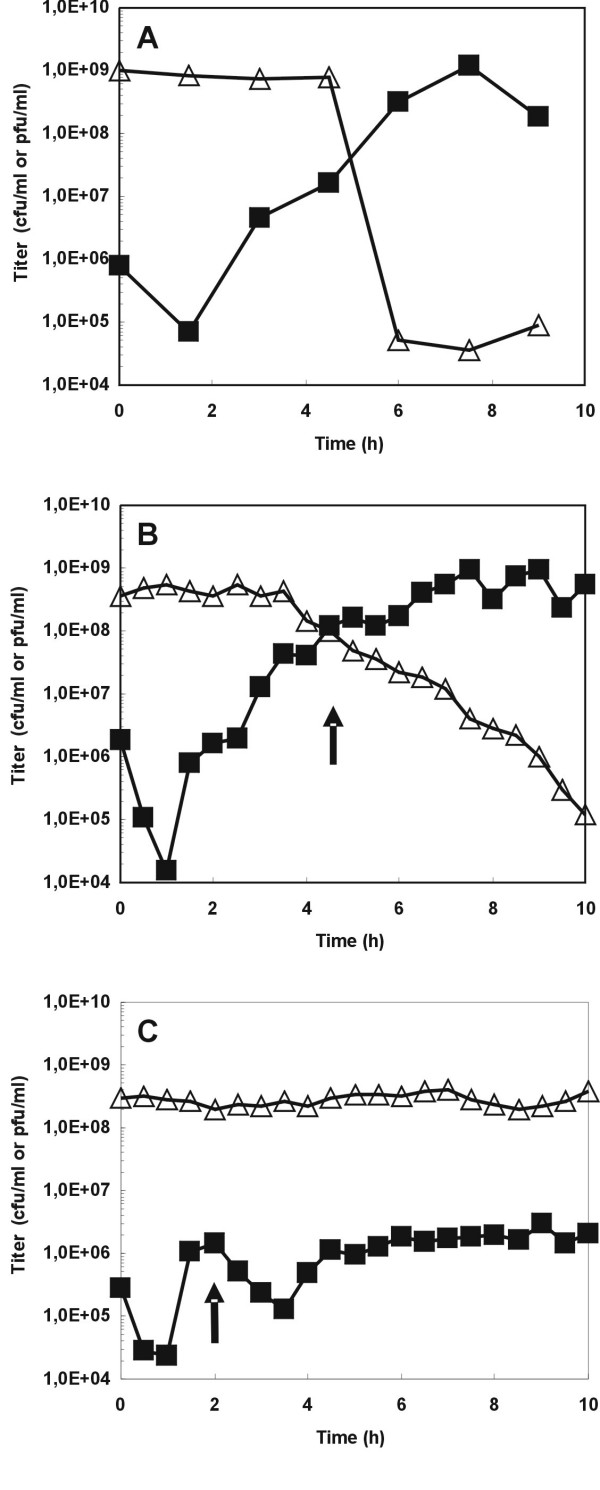
Development of bacteriophage P1 (strain P1*vir*) in *E. coli *MG1655 host growing in a chemostat under standard conditions (panel A), or when carbon source starvation was initiated (arrow) either 5 (panel B) or 2 (panel C) hours after infection (at m.o.i. = 0.01). Numbers of living bacterial cells (colony forming units) and phages (plaque forming units) are presented (open triangles and closed squares, respectively).

When *E. coli *cells cultured in a chemostat were infected by phage P1vir at low m.o.i. (0.01), they were destroyed in several hours, which was accompanied by rapid production of bacteriophage progeny (Fig. 2A). Deprivation of a carbon source at the time when absorbance of the bacterial culture started to decrease did not inhibit lytic development of the phage (Fig. 2B). However, such a development could be totally inhibited when starvation for a carbon source was initiated at early stages of phage development, i.e. relatively shortly (up to 2 hours) after infection (Fig. 2C).

**Figure 3:**
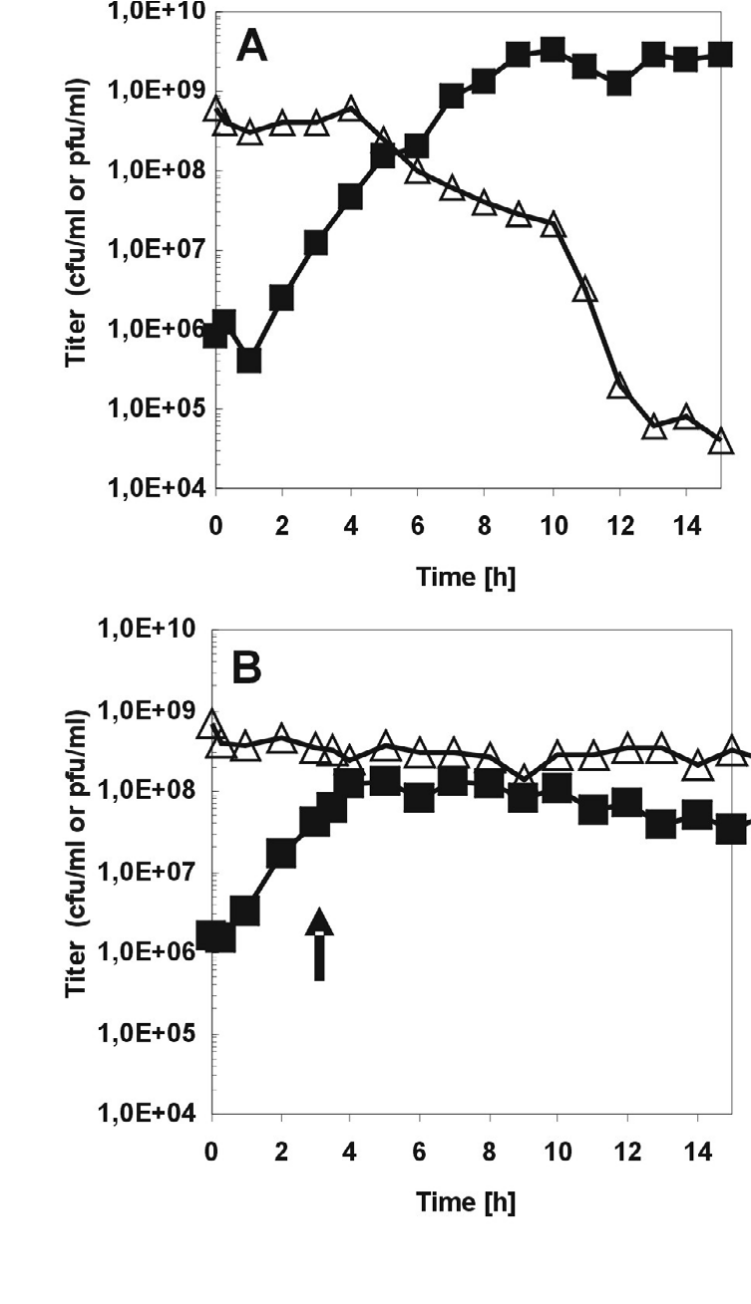
Development of bacteriophage T4 (strain T4D) in *E. coli *MG1655 host growing in a chemostat under standard conditions (panel A), or when carbon source starvation was initiated at the time indicated by arrow (3.5 h after infection at m.o.i. = 0.01) (panel B). Numbers of living bacterial cells (colony forming units) and phages (plaque forming units) are presented (open triangles and closed squares, respectively).

Similarly to bacteriophage P1, infection of bacterial cells growing in a chemostat with phage T4 resulted in its efficient development and devastation of the culture (Fig. 3A). This effect could be prevented by deprivation of the carbon source a few hours after infection (Fig. 3B).

## Discussion

We have demonstrated that development of bacteriophages λ, P1 and T4 can be completely inhibited after removing of a carbon source from the infected *E. coli *culture, provided that the starvation procedure is started at relatively early stages of phage lytic development. Therefore, to minimize deleterious effects of phage contamination, especially in high-cell density fed-batch cultivations, it may be recommended to stop feeding bacteria immediately after detection of first signs of phage infection.

Although unambiguous detection of phage contamination at early stages of infection may be difficult when using traditional methods, a newly developed technology of electric bio-chips allows for early detection of bacteriophages in bacterial cultures, even a few bacterial generations before they cause visible lysis of host cells [10-13]. One might also assume that, in endangered environments, phage propagation may be avoided by applying controlled low growth rates from the beginning of the cultivation process, and thus by avoiding batch phases with fast bacterial growth rates.

## Conclusion

Starvation of bacteriophage λ-, P1- or T4-infected *E. coli *cells results in severe inhibition of phage lytic development. This simple strategy cannot eliminate phages from the infected culture but it can ensure a dramatic decrease in phage burst size. Lower number of phages produced in contaminated bacterial cultures makes a contamination of the facility significantly less likely. Therefore, we believe that the method presented in this report may be useful in preventing phage spreading in fermentation facilities.

## Methods

### Bacteria and phages

*Escherichia coli *wild-type strain MG1655 [14] was used in all experiments. Bacteriophages λ papa, λ*cIb2*, P1vir and T4 were from our collection.

### Culture media and cultivation conditions

Luria-Bertani (LB) medium was as described previously [15]. Minimal media MM [16-18] and FB [19], containing various carbon sources, were employed. Bacteria were cultivated either in laboratory flasks or in a chemostat. In the latter case, the volume of the chemostat was 150 ml and the medium exchange rate was 30 mlmin^-1^. Carbon source deprivation was achieved by stopping the provision of nutrients by switching off the feed pump. Bacteria cultured in a chemostat were infected with bacteriophages after exchange of five volumes of the medium.

### Phage titration

Number of bacteriophages (plaque forming units, PFU) per volume unit was determined by standard titration. Briefly, 5 μl of serial dilutions of samples were spotted on bacterial lawn, prepared in a top, soft (0.7%) agar. Plaques were counted after overnight incubation at indicated temperature. Alternatively, 0.1 ml of each serial dilution of the sample was mixed with 0.1 ml of *E. coli *culture, incubated for 10 min, mixed with 3 ml of the top agar, and poured onto a standard LB plate. Plates were incubated as described above. In experiments with phage P1, CaCl_2 _was added to final concentration of 5 mM to allow efficient phage adsorption.

## Authors' contributions

MŁ conceived the study, participated in the design and coordination of the study, and participated in preparation of the manuscript, PG and JMŁ carried out chemostat experiments, AWJ carried out experiments with the phage development in flask cultures, AC participated in the design of flask cultures experiments, AW participated in the design of the study and helped to draft the manuscript, GW participated in the design and coordination of the study and drafted the manuscript, PN participated in the design and coordination of the study and helped to prepare the manuscript. All authors read and approved the final manuscript.
